# Kang Le Xin Reduces Blood Pressure Through Inducing Endothelial-Dependent Vasodilation by Activating the AMPK-eNOS Pathway

**DOI:** 10.3389/fphar.2019.01548

**Published:** 2020-01-22

**Authors:** Yixiu Zhao, Jiuxin Zhu, Hangfei Liang, Shuang Yang, Yannan Zhang, Weina Han, Chao Chen, Na Cao, Aruhan, Peiqiang Liang, Xing Du, Jian Huang, Jinhui Wang, Yan Zhang, Baofeng Yang

**Affiliations:** ^1^ State-Province Key Labratories of Biomedicine-Pharmaceutics of China, Key Laboratory of Cardiovascular Medicine Research, Ministry of Education, Department of Pharmacology, College of Pharmacy, Harbin Medical University, Harbin, China; ^2^ Department of Medicinal Chemistry and Natural Medicine Chemistry, College of Pharmacy, Harbin Medical University, Harbin, China

**Keywords:** hypertension, vascular endothelium, vasodilation, endothelial nitric oxide synthase, AMP-activate protein kinase

## Abstract

Hypertension is a major risk factor for stroke and cardiovascular events in clinic, which is accompanied by the abnormality of vascular tone and endothelial dysfunction of small artery. Here we report that Kang Le Xin (KLX), a novel anthraquinones compound, could reduce blood pressure and the underlying mechanisms involves that KLX induces endothelium-dependent vasodilation. KLX significantly decreases the arterial blood pressure of spontaneous hypertensive rats (SHR), decreases the contractile reactivity of superior mesenteric artery to phenylephrine and increases the vasodilatory reactivity of superior mesenteric artery to carbachol in a dose-dependent manner. Besides, KLX reduces vascular tension of endothelium-intact mesenteric artery pre-constricted with phenylephrine in a dose-dependent manner, while this effect is inhibited by depriving vascular endothelium or pretreating vascular rings with L-NAME (endothelial nitric oxide synthase inhibitor) or compound C (AMP-activated protein kinase inhibitor). Moreover, KLX increases nitric oxide (NO) generation, endothelial nitric oxide synthase (eNOS), AKT and AMP-activated protein kinase (AMPK) phosphorylation in cultured human umbilical vein endothelial cells (HUVECs), while these effects are inhibited by pretreating cells with compound C. In conclusion, KLX is a new compound with the pharmacological action of reducing arterial blood pressure. The underlying mechanism involves KLX induces endothelium-dependent vasodilation through activating AMPK-AKT-eNOS signaling pathway.

## Introduction

Hypertension is one of the most common cardiovascular diseases and a major risk factor for stroke and cardiovascular events in clinic (Judd and Calhoun, 2014). Studies have shown that improper management of hypertension causes 45% of stroke incidence and 50% of myocardial infarction incidence (Daniel et al., 2014; Emelia et al., 2017). In addition to blood vessels, hypertension also cause damage to heart, brain, kidney and other organs (Judd and Calhoun, 2014; Mills et al., 2016). Various factors can potentially induce hypertension, including genetic background, age, bad dietary habit and lifestyle (Calhoun et al., 2016). However, hypertension can be effectively controlled by anti-hypertensive drugs. Therefore, the study of anti-hypertensive drugs has extremely important clinical significance for controlling blood pressure and related cardiovascular events.

Studies have shown that the onset of hypertension is accompanied by the abnormality of vascular tone and endothelial dysfunction of small artery (Tykocki et al., 2018). Vascular endothelium lays on the inner surface of blood vessels. It senses normal and abnormal biological and mechanical signals in the blood, synthesizes and releases vasoactive substances, which induce the relaxation and contraction of vascular smooth muscle and affect vascular tension. The vasoactive substances released by the vascular endothelium fall into two categories: endothelium-derived relaxation factor (EDRF) and endothelium-derived contraction factor (EDCF) (Rubanvi, 1991; Tang and Vanhoutte, 2010). Besides, endothelium-derived hyperpolarization factor (EDHF), primarily intracellular K^+^, have been proposed as a novel vasoactive regulator of endothelium-dependent vasorelaxation. Intracellular K^+^ flows out through potassium channels of endothelial cells and VSMCs, hyperpolarizes cellular membrane potential and relaxes vascular smooth muscle (Busse et al., 2002). Nitric oxide (NO) is recognized as the most important EDRF. NO is a small molecule gas which can diffuse into vascular smooth muscle cells (VSMCs) and activate the Guanosine cyclase–cyclic GMP (GC-cGMP) signaling pathway to relax vascular smooth muscle. NO is mainly synthesized by endothelial nitric oxide synthase (eNOS) in vascular endothelial cells (Mount et al., 2007). eNOS is important to physiological processes that include neuronal signalling, inhibition of the hemostatic system, vasodilation and blood pressure control (Wood et al., 2013). Mice genetically deficient in eNOS (eNOS−/−) are hypertensive, indicating the importance of eNOS to blood pressure regulation and vascular homeostasis (Huang et al., 1995). A population-based study with Brazilian women showed that genetic polymorphisms of eNOS was significantly associated with a higher prevalence of hypertension, especially in older age and excess body weight groups, which further illustrated the closed correlation between endothelial eNOS and human hypertension (Neto et al., 2019). Multiple signaling pathways are involved in the regulation of eNOS activity. Phosphorylation is an important way to activate eNOS (Mount et al., 2007). Vascular endothelium perceives hemodynamic changes and metabolic stimulation signals and subsequently activates the related protein kinases, such as PI3K, CAMKII and AMPK to phosphorylate the terminal amino acid residue of eNOS (Zou et al., 2002; Datar et al., 2010; Gu et al., 2016). In addition to regulating vascular tension, NO released from endothelial cells can also inhibit platelet aggregation, adhesion of platelets and leukocytes to endothelial cells, generation of endothelin, and proliferation of VSMCs (Chatterjee et al., 2008). Therefore, the activity of eNOS and the release of NO can effectively protect vascular structure and function.

**Figure 1:**
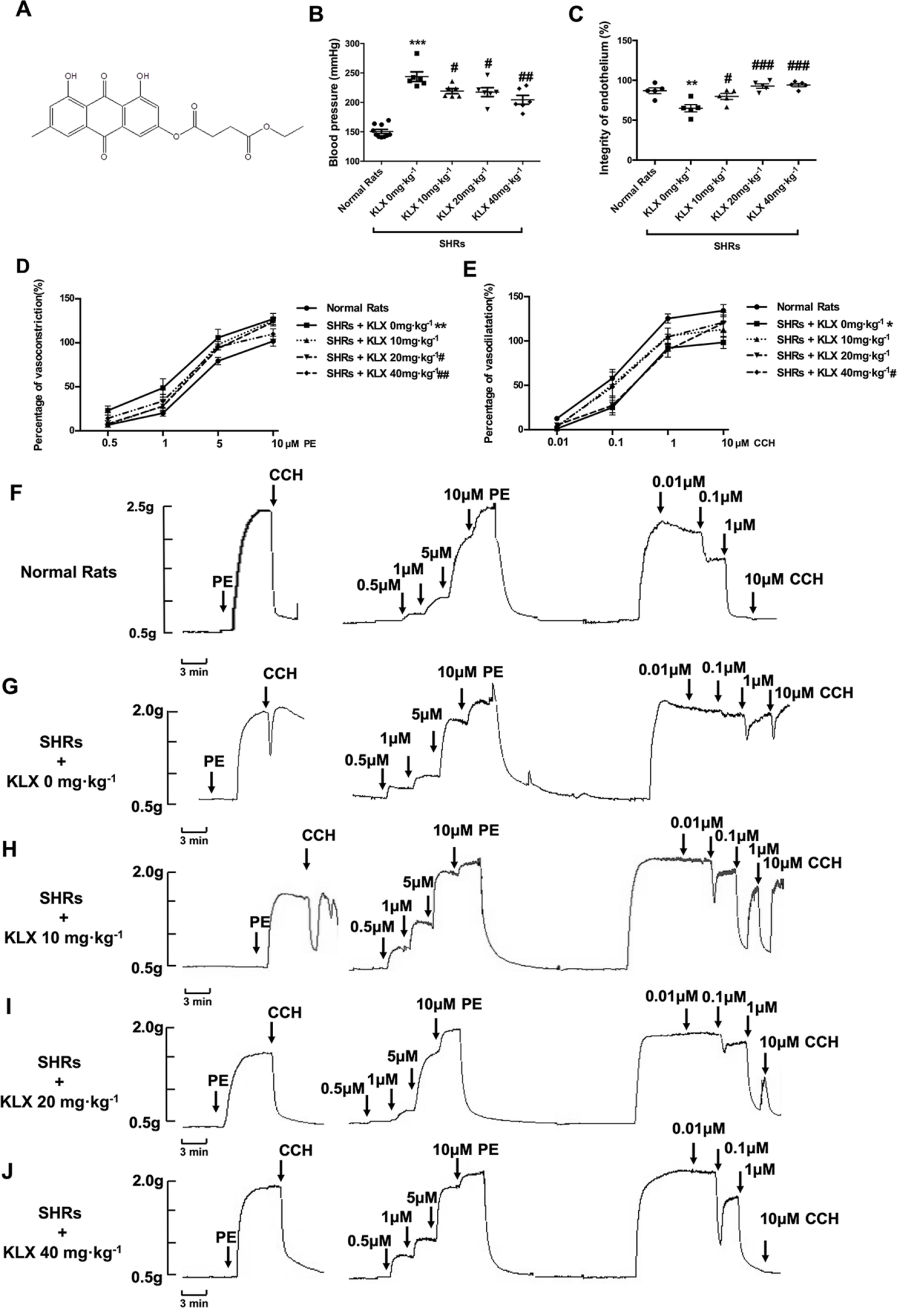
KLX reduces arterial blood pressure and corrects the abnormal contractility of superior mesenteric artery (SMA). **(A)** Chemical structure of KLX. **(B)** Oral administration with KLX 10, 20, 40 mg·kg^-1^ for 2 weeks significantly reduced arterial blood pressure. **(C)** Oral administration with KLX 10, 20, 40 mg·kg^-1^ for 2 weeks significantly protected endothelial integrity. **(D)** KLX reduced the increased contractile reactivity of SMA in SHR. **(E)** KLX increased the decreased vasodilatory reactivity of SMA in SHR. **(F)** Representative vascular tension recording image showed the SMA reactivity of control rats responded to different concentrations of PE or CCH. **(G)** Representative vascular tension recording image showed the alterations of SMA reactivity of SHR in Model group responded to different concentrations of PE or CCH. **(H)** Representative vascular tension recording image showed the effects of KLX 10 mg·kg^-1^on the SMA reactivity of SHR in response to different concentrations of PE or CCH. **(I)** Representative vascular tension recording image showed the effects of KLX 20 mg·kg ^-1^ on SMA reactivity of SHR in response to different concentrations of PE or CCH. **(J)** Representative vascular tension recording image showed the effects of KLX 40 mg·kg^-1^on SMA reactivity of SHR in response to different concentrations of PE or CCH. **(F**–**J)** are representative images of D and E. Data are presented as mean ± SEM, * *p* < 0.05, **p < 0.01, ***p < 0.001 *vs* Control; ^#^ *p* < 0.05, ^##^ *p* < 0.01, ^###^ *p* < 0.001 *vs* Model, n = 6.

Kanglexin (KLX) is a novel anthraquinone derivative which is designed and synthesized by Department of Medicinal Chemistry and Natural Medicine Chemistry of Harbin Medical University (**Figure 1A**). Many anthraquinone compound have hypotensive and vascular protective effects (Lim et al., 2014; Li et al., 2015). Whether KLX had hypotensive effects and whether KLX regulated vascular tension? This study aims to explore the pharmacological function of KLX on reducing arterial blood pressure and vascular tension, and to elucidate the underlying mechanisms.

## Materials and Methods

### Materials

KLX with a purity of 99% was provided by department of pharmaceutical chemistry (College of Pharmacy, Harbin Medical University). Spontaneous hypertensive rats (SHR, 8 weeks old) were purchased from Beijing Vital River Laboratory Animal Technology Co. (Beijing, China) (Gu et al., 2016). Carbachol (CCH), N′-Nitro-L-arginine-methyl ester hydrochloride (L-NAME), indomethacin (Indo), glibenclamide (Gliben), LY294002, compound C were purchased from Sigma-Aldrich Co. Ltd. (St Louis, USA). Phenylephrine (PE) and tetraethylammonium chloride (TEA) was purchased from Aladdin (Shanghai, China). EGTA was purchased from Solarbio Life Science (Beijing, China). Griess assay kit, DAF-FM DA fluorescent probe, bicinchoninic acid protein assay reagent kit (BCA kit) were purchased from Beyotime Biotechnology (Shanghai, China). Human umbilical vein endothelial cells (HUVECs) was purchased from Sciencell Research Laboratories (San Diego, CA, USA). (Beyotime, Shanghai, China). Primary antibodies for eNOS, phospho-eNOS ser1177, AMPKα1, phospho-AMPKα1, AKT, phospho-AKT antibodies were purchased from Cell Signaling Technology (Beverly, MA, USA). TBS buffer powder was purchased from Boster Biological Technology (Wuhan, China).

### *In Vivo* Studies

This study was carried out in accordance with the recommendations of US National Institutes of Health (NIH) guidelines for the care and use experimental animals. The protocol was approved by Ethics Committee of College of Pharmacy, Harbin Medical University. SHRs (8 weeks old) were divided into four groups according to their initial blood pressure measured by tail blood pressure meter (BP2010, Softron, Beijing, China), including KLX 0 mg·kg^-1^ (SHR without KLX administration), KLX 10 mg·kg^-1^(SHR administrated with KLX 10 mg·kg^-1^·d^-1^), KLX 20 mg·kg^-1^(SHR administrated with KLX 20 mg·kg^-1^·d^-1^), and KLX 40 mg·kg^-1^ (SHR administrated with KLX 40 mg·kg^-1^·d^-1^) groups. Normal Sprague Dawley (SD) rats served as the control group (Normal Rats). Rats were administrated with KLX by gavage daily for consecutive 2 weeks. Following the final administration, the rats were anesthetized, and their common carotid artery was intubated and connected to a pressure transducer for measuring arterial blood pressure (BL-420S, Techman, Chengdu, China).

### Measurement of Vascular Tension

Vascular tension of mesenteric artery was examined by microvascular tension measurement system (Danish Myo Technology, Denmark) according to the published method with minor modifications (Zhao et al., 2016). Firstly, SHRs were anesthetized and their superior mesenteric arteries were dissected out and placed into pre-cooled and oxygenated Krebs–Henseleit (K-H) buffer containing (in mM): NaCl 118, NaHCO_3_ 25, D-glucose 11, KCl 4.7, KH_2_PO_4_ 1.2, MgSO_4_ 1.17, and CaCl_2_ 2.5, pH 7.4. Perivascular adipose tissue was removed and the vessel was carefully cut into several rings of 2-mm in length. In another set of experiments, the inner wall of vessels was scratched using forceps to destroy the integrity of the vascular endothelium. Then the vascular rings were perfused in Krebs solution aerated with 95% O_2_ and 5% CO_2_ at 37°C. The vascular tone was adjusted to and stabilize at 0.5 g. The functional integrity of endothelium was examined by using 20 μM PE to constrict the ring and 10 μM CCH to relax it. The integrity of endothelium was recorded and vascular rings were washed out with K-H buffer to its basal tension state. Then PE was added cumulatively in the organ bath, with the final concentration of 0.5 µM, 1 µM, 5 µM, 10 µM and the interval time of 3 minutes, to detect the response of vascular rings to PE – induced vasoconstriction. Then the vascular rings were washed out with K-H buffer again to its basal tension state and CCH was added cumulatively in the organ bath, with the final concentration of 0.01 µM, 0.1 µM, 1 µM, 10 µM and the interval time of 3 minutes, to detect the response of vascular rings to CCH - induced vasorelaxation. In another series of experiment performed on normal SD rats, the integrity of vascular endothelium was recorded and endothelial intact vascular rings were screened out (The rings with >80% of the full relaxation were considered as endothelium-intact, while the rings with <20% relaxation were considered as endothelium-denuded). After the vascular rings had been constricted with PE, KLX was added cumulatively in the organ bath and the vascular tension was monitored. In separate experiments, vascular rings were incubated with L-NAME 100 μM, Indo 10 μM, Gliben 10 μM, TEA 1 mM, LY294002 10 μM, compound C 10 μM and EGTA 200 μM for 15 min before being constricted with PE.

### Determination of NO Release

NO concentration was determined by Griess assay kit and DAF-FM DA fluorescent probe in cultured HUVECs. For Griess assay, HUVECs were seeded on 96-well plate and incubated with different concentrations of KLX for 5, 10, 20, and 40 min. Then the culture medium was collected at different time points and the concentration of NO was determined by measuring total nitrites with a Griess assay kit. The concentration of nitrite was determined by visible spectrophotometry at 540 nm and calculated through a standard curve derived from NaNO2 (0–100 μM). For DAF-FM DA determination, the culture medium was discarded and the cells were loaded with DAF-FM DA fluorescent probe according to the instruction for determining NO concentration (Yu et al., 2012).

### Western Blot Analysis

Protein concentrations were determined by Western blot analysis. Specifically, HUVECs were incubated with KLX 90 μM for 10 min before lysing with ice-cold lysis buffer containing protease and phosphatase inhibitor. Then the lysate was centrifuged at 13,500 g at 4°C for 15 min, and the supernatant was collected for determining the protein concentration using BCA kit. The samples were separated by electrophoresis on 12% or 10% sodium dodecyl sulfate polyacrylamide gel and transferred onto nitrocellulose membranes. Residual protein bands were blocked with 5% milk-TBS buffer. The membranes were incubated with rabbit anti-eNOS/p-eNOS (1:1000), anti-AMPK/p-AMPK (1:1000), and anti-AKT/p-AKT antibodies (1:1000) diluted in phosphate buffered saline (PBS). Next, the membranes were incubated with HRP-conjugated, secondary anti-rabbit antibody at a 1:10000 dilution in PBS. Protein levels were determined using the Odyssey Imaging System (LI-COR Biosciences, Lincoln, NE, USA). β-actin (1:4000) was used as an internal reference for data normalization.

### Data and Statistical Analysis

All data are presented as mean ± SEM. Relaxation of vascular rings was expressed as a percentage of decreasing vascular tension to the maximum contractile response induced by PE. Results were analyzed by using paired *t*-test and ANOVA analysis. When P value was less than 0.05, data between two groups was considered to be statistical difference. All original data are provided in the supplementary data (**Supplementary Materials**).

## Results

### KLX Reduces Arterial Blood Pressure of SHR and Corrects the Abnormal Contractility of Superior Mesenteric Artery

As anticipated, the blood pressure of SHR without KLX administration (SHR + KLX 0 mg·kg^-1^) group was 243.79 ± 20.01 mmHg, which was significantly higher than that of the Normal Rats group 149.26 ± 10.78 mmHg. After 2 weeks of KLX treatment, the blood pressure of SHRs in KLX 10, 20, 40 mg·kg^-1^ group were 219.19 ± 10.37, 217.55 ± 18.58, 204.40 ± 18.26 mmHg, which were all significantly lower than SHRs in KLX 0 mg·kg^-1^ group (**Figure 1B**).

Meanwhile, the superior mesenteric artery (SMA) of SHR without KLX administration group had a poor endothelium integrity compared with normal rats. After KLX treatment, endothelium integrity was significant improved, suggesting that KLX had endothelial protective effect (**Figure 1C**). Besides, the SMA of SHRs in KLX 0 mg·kg^-1^ group had a poor endothelium-dependent vasodilation, manifesting as the increased contractive reactivity to PE and a decreased vasodilatory reactivity to CCH compared with normal rats, indicating the enhanced arterial constriction associated with hypertension (**Figures 1D, E** are statistical results of SMA reactivity responded to PE or CCH; **Figures 1F, G** are representative images of SMA reactivity of normal rats and SHRs administrated without KLX responded to PE or CCH respectively). While these alterations were effectively mitigated by KLX administration. SHRs with KLX 20, 40 mg·kg^-1^ administration had a significantly decreased PE - induced SMA vasoconstriction compared with SHRs without KLX administration; SHRs with KLX 40 mg·kg^-1^ administration had a significantly enhanced CCH - induced SMA vasodilation compared with SHRs without KLX administration (**Figures 1D, E** are statistical results of SMA reactivity responded to PE or CCH; **Figures 1H–J** are representative images of SMA reactivity of SHRs administrated with KLX 10, 20, 40 mg·kg^-1^ responded to PE or CCH respectively).

### KLX Reduces Vascular Tension of Endothelium-Intact Artery Through Activating eNOS and Increasing NO Release

**Figure 2:**
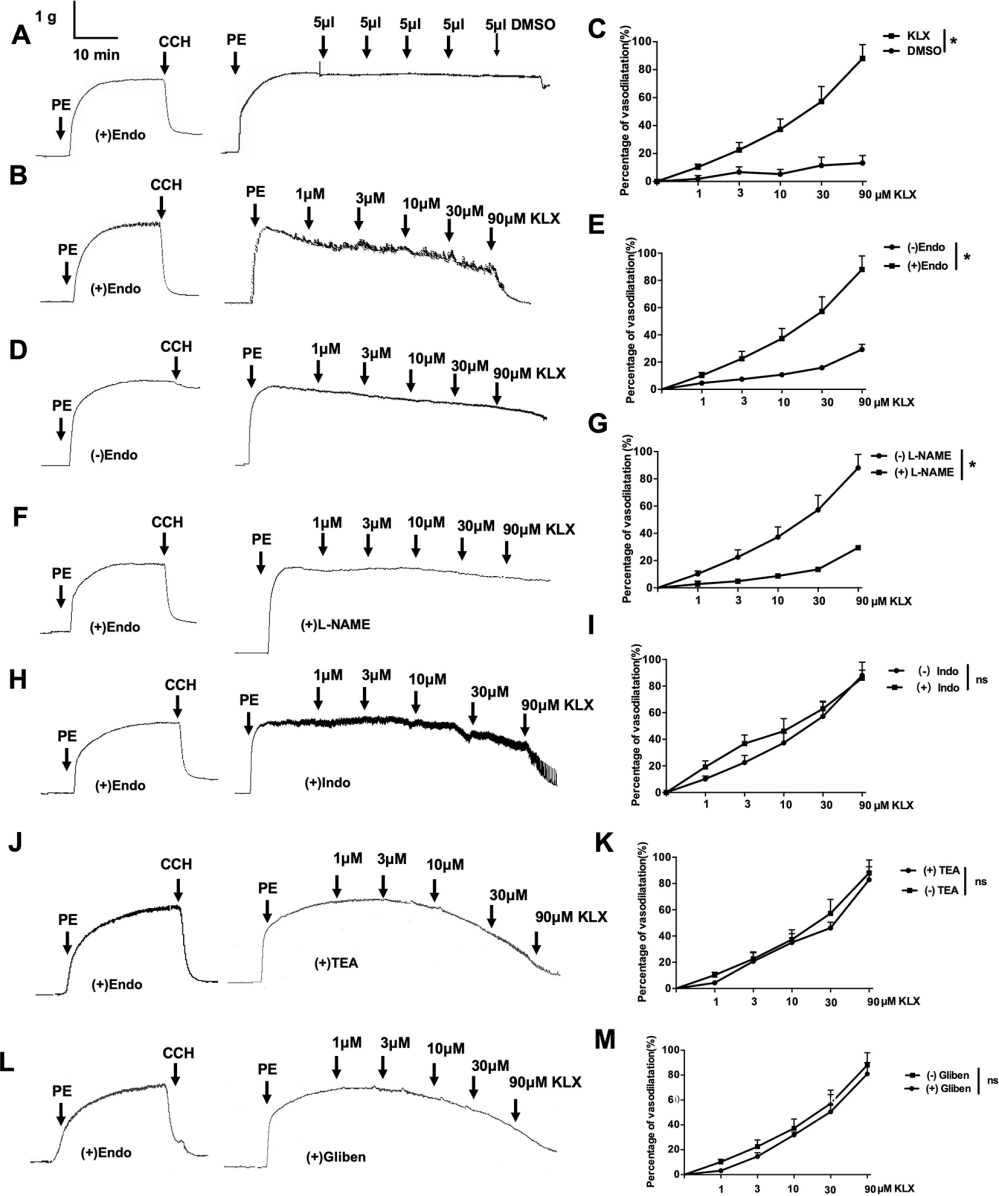
The vascular tension-relieving effects of KLX depend on endothelial integrity and NO generation. **(A)** Lack of effect of DMSO on the tension of endothelium-intact vascular rings. **(B)** Relieving effects of varying concentrations (1, 3, 10, 30, and 90 µM) of KLX on the tension of endothelium-intact vascular rings. **(C)** Dose-response curves of the effects of KLX and DMSO on the tension of endothelium-intact vascular rings. **(D)** Effect of KLX on the tension of endothelium-denuded vascular rings. **(E)** Dose-response curves of the effect of KLX on the tension of endothelium-intact and endothelium-denuded vascular rings. **(F)** Inhibitory effect of L-NAME (eNOS inhibitor) on the vasodilating effect of KLX. **(G)** Comparison of the effects of KLX on the tension of endothelium-intact vascular rings with or without pretreatment of L-NAME. **(H)** Effect of indometacin (Indo: COX inhibitor) on the vasodilating effect of KLX. **(I)** Comparison of the effects of KLX on the tension of endothelium-intact vascular rings with or without pretreatment of indometacin. **(J)** Effect of TEA (K_Ca_ inhibitor) on the vasodilating effect of KLX. **(K)** Comparison of the effects of KLX on the tension of endothelium-intact vascular rings with or without pretreatment of TEA. **(L)** Effect of glibenclamide (Gliben: K_ATP_ inhibitor) on the vasodilating effect of KLX. **(M)** Comparison of the effects of KLX on the tension of endothelium-intact vascular rings with or without pretreatment of Gliben. Data are presented as mean ± SEM, *p < 0.05; ns, no significance; n = 5.

The data presented above indicate that KLX elicits anti-hypertensive effect likely by maintaining normal contractility of SMA. To further delineate the molecular mechanisms by which KLX improves arterial vascular tone, we isolated SMA from normal SD rats and prepared endothelium-intact or endothelium-denuded vascular rings. Next, we examined the effects of KLX on PE-induced vasoconstriction. As illustrated in **Figures 2A–C**, KLX reduced the tension of endothelium-intact vascular rings in a dose-dependent manner, whereas as the solvent control, cumulative additions of DMSO up to a final concentration of 2.5‰ did not elicit any significant effect. However, depriving vascular endothelium significantly inhibited KLX-induced vasodilation, suggesting that the vasodilating effect of KLX was mediated by vascular endothelium (**Figures 2D, E**).

Moreover, L-NAME (eNOS inhibitor) pretreatment significantly inhibited KLX-induced vasodilation of endothelium-intact vascular rings (**Figures 2F, G**), while indometacin (COX inhibitor), TEA (K_Ca_ inhibitor) and glibenclamide (K_ATP_ inhibitor) pretreatment had no such an effect (**Figures 2H–M**), suggesting that KLX-induced vasodilation depends on eNOS activity and NO availability in vascular endothelium.

**Figure 3:**
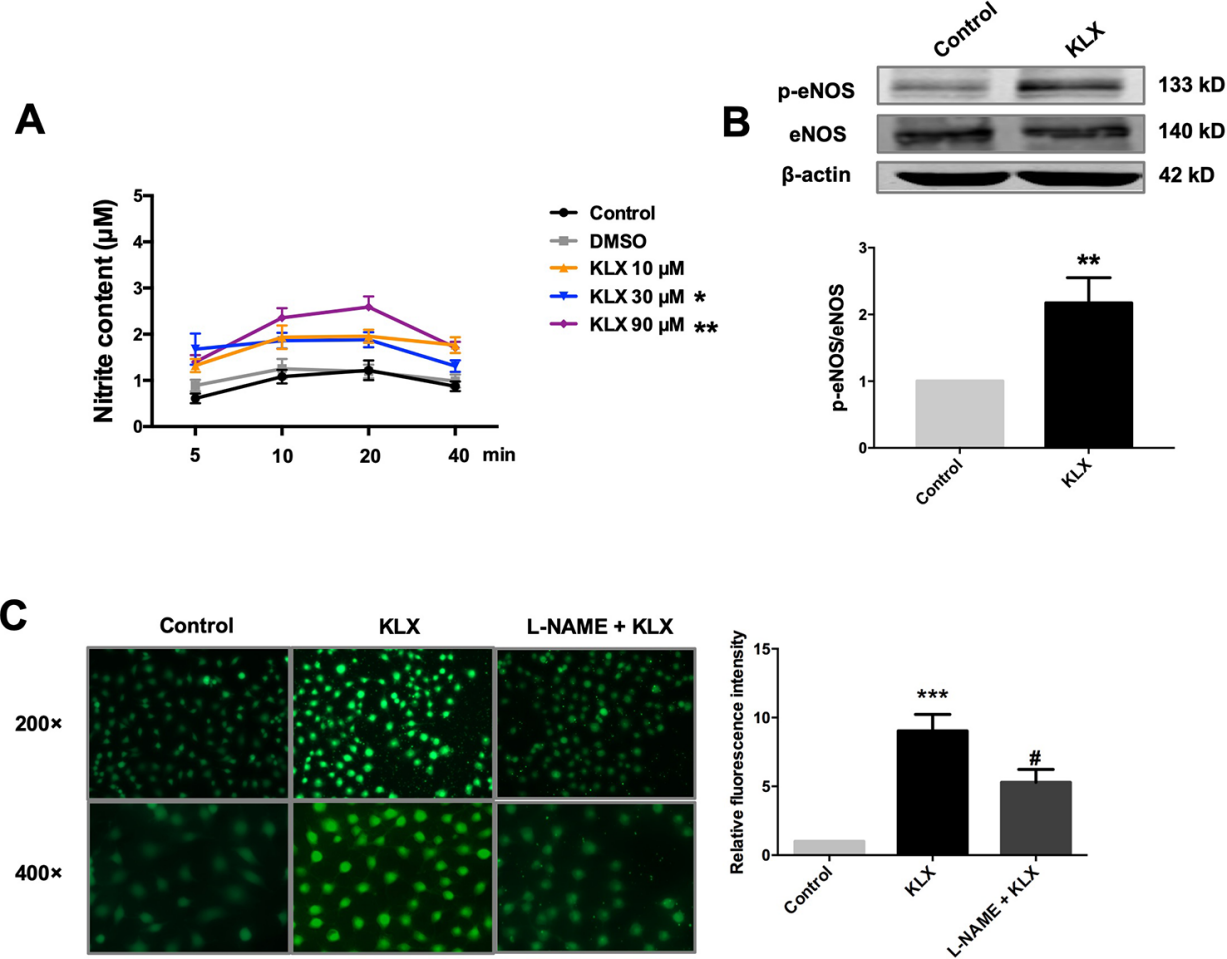
KLX enhances phosphorylation of eNOS and production of NO. **(A)** Promoting effect of KLX on NO production in cultured HUVECs. HUVECs were pretreated with different concentrations of KLX (10, 30, 90 µM) for 5, 10, 20, 40 min respectively. Then the content of nitrite (which represents NO concentration) in cell culture medium of different incubation time and different KLX concentrations was determined using Griess reagent assay. Data were presented as the relative value by taking the nitrite content of control group at different time points as a reference, *p < 0.05, **p < 0.01 vs control, n = 6. **(B)** Enhancing effect of KLX on eNOS phosphorylation in cultured HUVECs. **p < 0.01 *vs* control, n = 6. **(C)** Representative images (left panel) and statistical data (right) of fluorescence staining of NO showing the suppressing effect of L-NAME (a NOS inhibitor) on KLX-induced NO production in cultured HUVECs. Data are presented as mean ± SEM, ***p < 0.001 *vs* control, ^#^ *p* < 0.05 *vs* KLX, n = 5.

Furthermore, in cultured HUVECs, KLX (90 μM) significantly increased NO generation and eNOS phosphorylated activation (p-eNOS ser1177), and these effects were abrogated when the cells had been pretreated with L-NAME (**Figure 3**). These results indicate that KLX reduced vascular tension through activating eNOS and increasing NO release.

### KLX Activates eNOS and Increases NO Release Through Activating the AMPK-AKT Pathway

**Figure 4:**
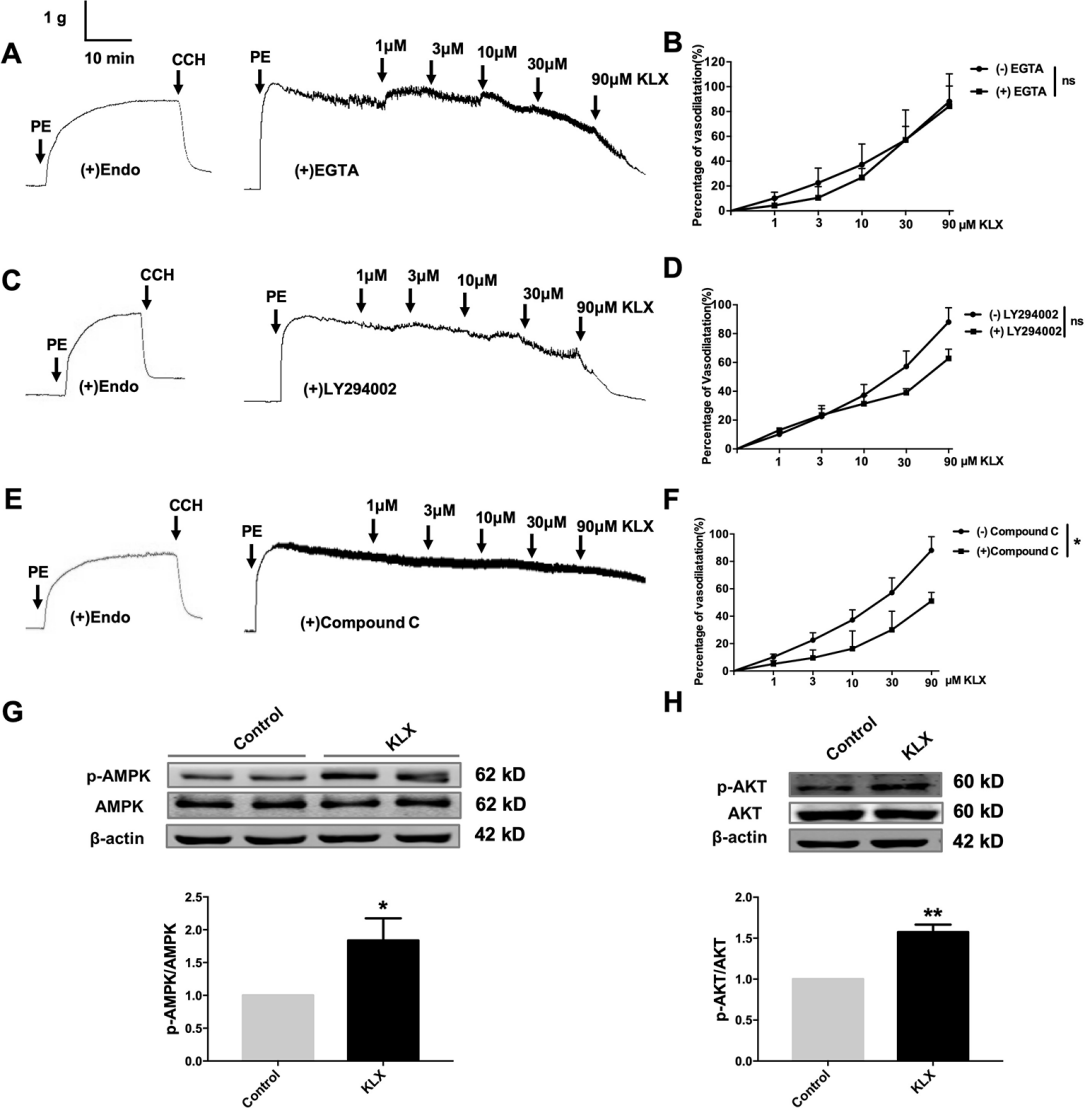
KLX reduces vascular tension of endothelium-intact artery through activating AMPK-AKT pathway. **(A)** Lack of effect of EGTA (CAMKII inhibitor; 200 µM on the vasodilating effect of KLX. **(B)** Dose response curves of the effect of KLX on the tension of endothelium-intact vascular rings with or without pretreatment of EGTA. ns, no significance, n = 5. **(C)** Lack of effect of LY294002 (PI3K inhibitor; 10 µM) on the vasodilating effect of KLX. **(D)** Dose response curves of the effect of KLX on the tension of endothelium-intact vascular rings with or without pretreatment of LY294002. ns, no significance, n = 5. **(E)** Inhibitory effect of compound C (AMPK inhibitor; 10 µM) on the vasodilating effect of KLX. **(F)** Dose response curves of the effect of KLX on the tension of endothelium-intact vascular rings with or without pretreatment of compound C. *p < 0.05, n = 5. **(G)** Enhancing effect of KLX on AMPK phosphorylation in cultured HUVECs, as indicated by the increase in the phosphorylated/activated form of AMPK (p-AMPK). *p < 0.05 *vs* control, n = 8. **(H)** Enhancing effect of KLX on AKT phosphorylation in cultured HUVECs, as indicated by the increase in the phosphorylated/activated form of AKT (p-AKT). Data are presented as mean ± SEM, **p < 0.01 *vs* control, n = 6.

It is known that eNOS can be phosphorylated by various protein kinases in endothelial cells, such as PI3K, CAMKII and AMPK. In order to delineate which of these kinases mediates the vasodilating and eNOS-activating effects of KLX, we conducted the following experiments. We first demonstrated that pretreatment with neither EGTA (CAMKII inhibitor, 200 μM) nor LY294002 (PI3K inhibitor, 10 μM) affected the vasodilating effect of KLX (**Figures 4A–D**), while compound C (AMPK inhibitor, 10 μM) significantly inhibited KLX-induced vasodilation (**Figures 4E, F**).

We then further observed that KLX significantly increased phosphorylation of AMPK (p-AMPK) and AKT (p-AKT; **Figures 4G, H**).

**Figure 5:**
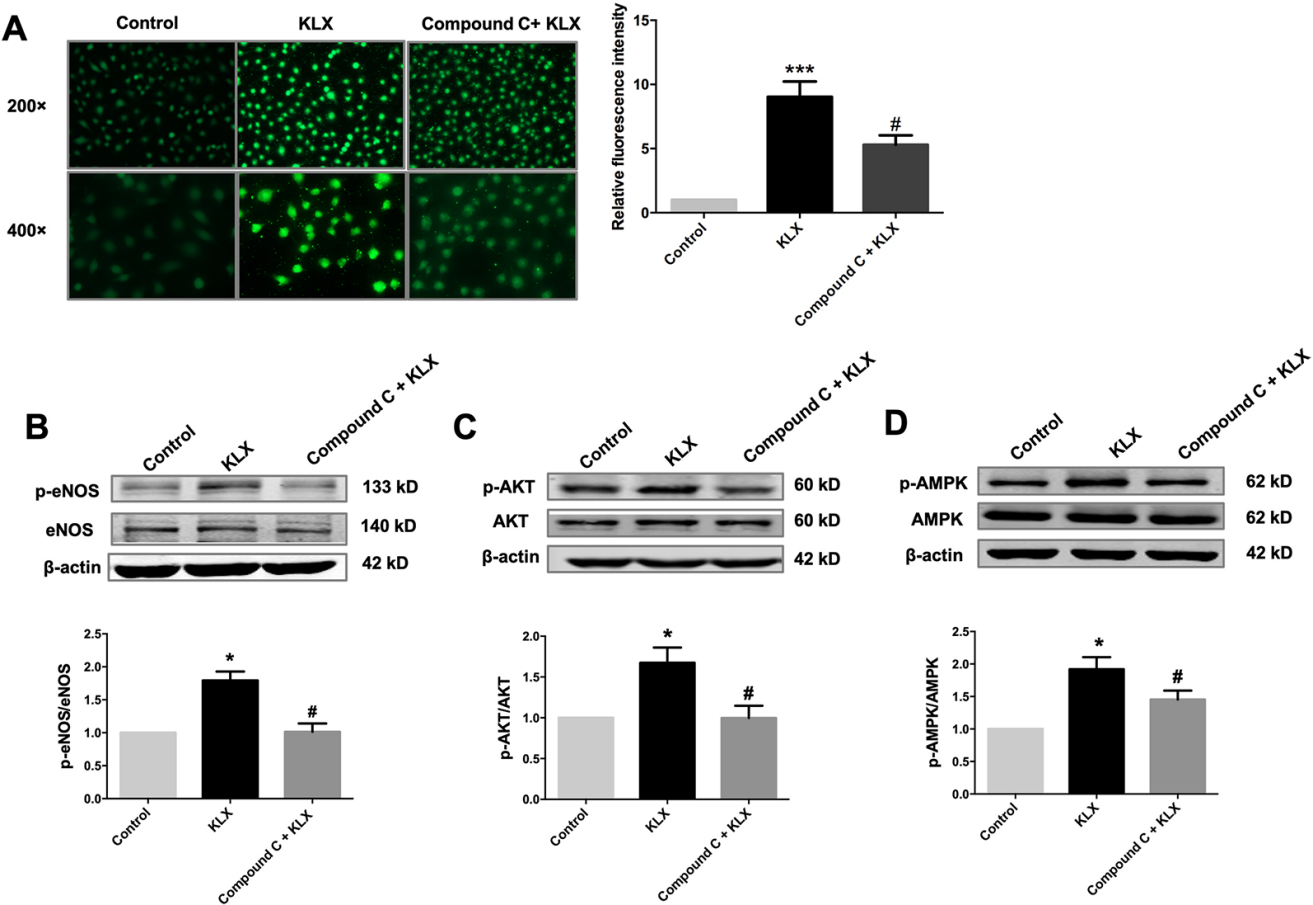
KLX increases NO release through activating the AMPK-AKT pathway. **(A)** Typical examples (left panel) and statistical data (right) of DAF-FM DA fluorescent staining of NO showing the inhibitory effect of compound C (10 µM) on KLX-induced NO production in HUVECs. ***p < 0.001 *vs* control, #*p* < 0.05 *vs* KLX, n = 5. **(B)** Inhibitory effect of compound C on KLX-induced phosphorylation of eNOS protein. *p < 0.05 *vs* control, ^#^ *p* < 0.05 *vs* KLX, n = 6. **(C)** Inhibitory effect of compound C on KLX-induced phosphorylation of AKT protein. *p < 0.05 *vs* control, ^#^ *p* < 0.05 *vs* KLX, n = 6. **(D)** Inhibitory effect of compound C on KLX-induced phosphorylation of AMPK. *p < 0.05 *vs* control, ^#^ *p* < 0.05 *vs* KLX; n = 8.

Next, we showed that compound C significantly inhibited KLX-induced production of NO and phosphorylated activation of AMPK, AKT, and eNOS, suggesting that inhibition of AMPK attenuated KLX-induced activation of AMPK, AKT, eNOS and NO generation. Therefore, these results indicated that KLX activated eNOS and increased NO release through activating the AMPK-AKT pathway (**Figure 5**).

## Discussion

Hypertension is an independent risk factor for acute cardiac and cerebral vascular events such as stroke and acute myocardial infarction. If not controlled effectively, hypertension can damage multiple organs such as heart, brain and kidney. Therefore, exploring the pathophysiological mechanisms and developing drugs for treatment of hypertension is important for both patients and clinicians (Park et al., 2015; Sim et al., 2015; Lobo et al., 2017). We demonstrated that KLX effectively lowered down the high arterial blood pressure in SHR, a commonly used animal model for studying the pathogenesis of hypertension and evaluating anti-hypertensive drugs (Spitler et al., 2013; Marina et al., 2015). However, in our study, the blood pressure data seems a little high both in the normal animals and 8 weeks SHR rats. We think it may be caused by the pressure transducer in our experimental system. However, it can be seen from the data presented in the results that the blood pressure values of animals are stable. Besides, the blood pressure of SHR group is significantly higher than Normal rats group, confirming that the blood pressure data are stable and reliable. In conclusion, we proved the pharmacodynamic effect of KLX on lowering blood pressure *in vivo* experiments. Next, we explored the pharmacological mechanism of KLX on lowering blood pressure.

Studies have shown that hypertensive patients are often accompanied by abnormal increases in vascular tone of small arteries (Toth et al., 2013; Brozovich et al., 2016). Artery is a biological soft tissue with viscoelasticity, which can adjust its own tension according to changes of neuro-hormonal factors in the living system, affect the lumen size and the blood flow in blood vessels, and regulate blood pressure and hemodynamics of circulation system (Tykocki et al., 2018). Clinical studies have found that the occurrence of hypertension is closely associated with dysregulation of arterial vascular tension that causes abnormal contraction of blood vessels (Bernatova, 2014; Touyz et al., 2018). Therefore, maintaining the elasticity of the blood vessel wall, regulating the vascular tone, and avoiding abnormal contraction of blood vessels under stress conditions are of great significance for treating hypertension. We showed here that the superior mesenteric arteries of SHR exhibited greater contractility in response to PE-induced vasoconstriction and lower vasodilatory reactivity in response to CCH-induced vasorelaxation compared with normal control rats. Notably, KLX effectively corrected these detrimental alterations. In addition, we have also demonstrated that KLX reduced the tension of vascular rings pre-constricted by PE in a dose-dependent manner. However, the relationship between KLX dose and plasma concentration was unclear now and it cannot be excluded that other mechanisms may contribute to the blood pressure lowering effect of KLX. Further studies will explore other potential mechanisms of KLX on lowering blood pressure.

The vascular endothelium locates on the inner surface of the arterial wall, which regulates multiple pathophysiological processes by perceiving hemodynamic changes and neuro-hormonal signals (Gimbrone and García-Cardeña, 2016). Research showed that vascular endothelium secretes various vasoactive substances to regulate vascular tension and affect blood pressure, coagulation and anticoagulant factors to regulate thrombosis, inflammatory factors to regulate vascular inflammatory response and atherosclerosis, and angiogenic factors to regulate angiogenesis and vascular injury repair (Chiu and Chien, 2013; Rajendran et al., 2013; Wood et al., 2013; Johnson and Wilgus, 2014). Under normal conditions, vascular endothelial cells regulate vascular tone by synthesizing a balanced mount of EDRF and EDCF. Under pathological conditions, vascular endothelial dysfunction occurs and the ability of endothelial cells to synthesize EDRF is impaired, resulting in enhanced ability of EDCF to contract blood vessels and trigger excessive vasoconstriction(Giles et al., 2012; Kang, 2014). Hence, synthesis of EDRF is the basis for ensuring the physiological function of arterial vascular endothelium and the regulation of arterial vascular tension. Therefore, drugs that can increase EDRF synthesis are important for protecting arterial endothelial function and inhibiting abnormal arterial vasoconstriction (Stankevicius et al., 2003; Dudzinski et al., 2006). In this study, we found KLX reduced the tension of endothelium-intact vascular rings pre-constricted with PE, while this reduction was significantly inhibited by destroying vascular endothelium, indicating that the vasodilating effect of KLX was endothelium-dependent.

NO and PGI_2_ are the most important EDRFs in vascular endothelial cells. In endothelial cells, NO is mainly synthesized through regulation the function of eNOS which catalyzes its substrate L-arginine and coenzyme BH_4_, and PGI_2_ synthesis is primarily catalyzed by COX. NO diffuses into VSMC and activates the GC-cGMP signaling pathway to induce vasodilation, and PGI_2_ binds to its receptor on the membrane of VSMC and activates AC-cAMP-PKA signaling pathway to induce vasodilation (Kukovetz et al., 1979; Dudzinski et al., 2006). We found here that the vasodilating effect of KLX depended on endothelial NO rather than PGI_2_, as indicated by the results that KLX-induced vasodilation was inhibited by eNOS inhibitor L-NAME, but not by COX inhibitor indometacin. Meanwhile, KLX promoted NO production in cultured HUVECs, further supporting that KLX-induced vasodilation relies on its promotion of NO production.

Except for NO and PGI_2_, several studies show that endothelium-derived hyperpolarization is the most important vasodilator mechanism in small arteries and hence of importance for blood pressure. Opening of potassium channels on vasculature with resultant hyperpolarization is an fundamental mechanism of endothelium-derived hyperpolarization. Calcium-activated potassium channel (K_Ca_) and ATP-sensitive potassium channel (K_ATP_) are major targets for vasoactive substances to induce membrane hyperpolarization. Besides, K_Ca_ and K_ATP_ channel also participate in stabilizing membrane potential and regulating cellular membrane excitability according to intracellular Ca^2+^ and ATP concentration (Zhao et al., 2016). Hence, the involvement of K_Ca_ and K_ATP_ channel in KLX-induced vasodilation was examined. Our results showed that TEA (K_Ca_ inhibitor) and glibenclamide (K_ATP_ inhibitor) had no significant effect on KLX induced endothelium-dependent vasodilation. Therefore, we speculated that KLX did not lead to endothelial hyperpolarization. Besides, effect of KLX on the tension of arteries with smaller lumen was not investigated in this study, which was a limitation of our study, since microcirculation play an important role in the regulation of peripheral vascular resistance (Fenger-Gron et al., 1997). However, the present study well showed that KLX decreased blood pressure and increased NO-mediated vasorelaxation in rat superior mesenteric artery.

**Figure 6:**
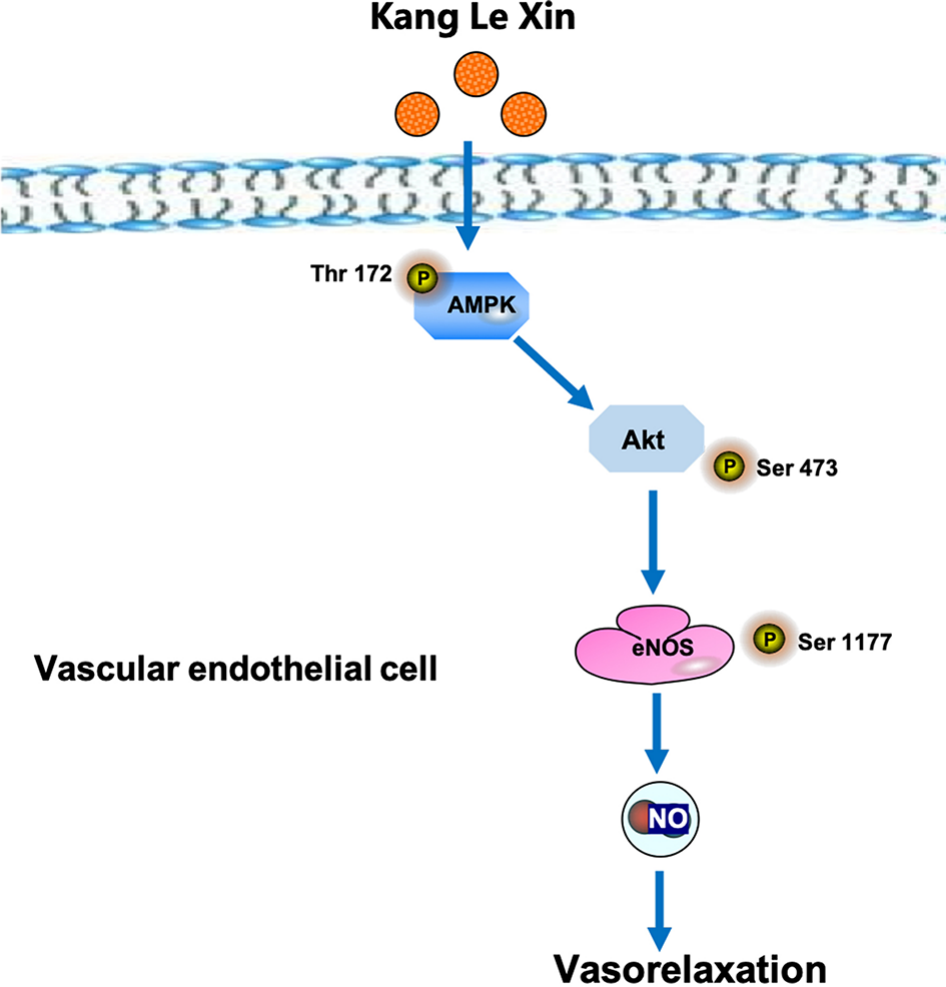
Schematic diagram of the proposed signaling mechanisms by which KLX produces hypotensive effect.

Serine phosphorylation is an important way to regulate eNOS activity in endothelial cells (Govers and Rabelink, 2001). Our results demonstrated that KLX increased the protein abundance of p-eNOS ser-1177 in HUVECs, suggesting that the increase in the biosynthesis of NO is likely due to the enhanced activation of eNOS through phosphorylation on the serine-1177 amino acid residue. ENOS can be activated by multiple signaling pathways, which can be roughly divided into the calcium-dependent pathway and the non-calcium-dependent pathway. In the case of calcium-dependent eNOS activation, an increase of Ca^2+^ in vascular endothelial cells activates CaM and CaMKII, which subsequently phosphorylates eNOS and increases NO generation (Tran et al., 2000). Endothelial cells can perceive blood flow shear stress or circulating mechanical stimulation signal and activate protein kinase PI3K; in addition, endothelial cells can also sense metabolic stimulation signals and activate protein kinase AMPK. Both PI3K and AMPK can activate downstream protein AKT and phosphorylate eNOS at its terminal residue serine-1177 (Govers and Rabelink, 2001; Ning and Zhao, 2013). We observed that inhibiting AMPK activities significantly reduced the vasodilating effect of KLX, while inhibiting intracellular Ca^2+^ and PI3K activity had no such an effect, indicating that KLX-induced vasodilation is dependent on AMPK, AKT and eNOS activities. Besides, KLX significantly increased the expression of p-AMPK and p-AKT, and KLX-increased phosphorylation of AKT and eNOS and production of NO as well were significantly inhibited by inhibiting AMPK activity. These findings strongly suggest that KLX induces vasodilation through activating the AMPK-AKT-eNOS signaling pathway (**Figure 6**).

## Conclusion

The present study provided strong evidence suggesting that KLX is an anti-hypertensive agent that works by causing vasodilation through activating the AMPK-AKT-eNOS/NO signaling pathway. Our findings thus allow for novel insight into the pharmacological properties of KLX and a molecular/signaling mechanism underlying the beneficial effects of KLX.

## Data Availability Statement

All datasets generated for this study are included in the article/**Supplementary Data Sheet 1**.

## Ethics Statement

This study was carried out in accordance with the recommendations of US National Institutes of Health (NIH) guidelines for the care and use experimental animals. The protocol was approved by the Ethics Committee of College of Pharmacy, Harbin Medical University.

## Author Contributions

YaZ and BY contributed to project administration and supervision. YxZ and JZ contributed to original draft preparation. HL and SY contributed to determine the blood pressure and vascular tension. PL, YnZ, and XD contributed to cell culture and molecular biology experiment. CC and NC conducted supplementary experiments, collected and processed supplementary data. WH and A prepared the required compound. JH and JW designed the compound. All authors approved the submitted version of manuscript.

## Funding

This work was supported by China Postdoctoral Science Foundation Grant (NO. 2018M641882), National Natural Science Foundation of China (NO. 81870259, NO. 81730012 and NO. 81861128022), Heilongjiang Postdoctoral Foundation (NO. LBH-Z18123).

## Conflict of Interest

The authors declare that the research was conducted in the absence of any commercial or financial relationships that could be construed as a potential conflict of interest.
